# Antimicrobial resistance in chronic liver disease

**DOI:** 10.1007/s12072-019-10004-1

**Published:** 2019-12-03

**Authors:** Vishal C. Patel, Roger Williams

**Affiliations:** 1 The Institute of Hepatology London and Foundation for Liver Research, 111 Coldharbour Lane, London, SE5 9NT UK; 2 The Institute of Liver Studies and Transplantation, King’s College Hospital NHS Foundation Trust, Denmark Hill, London, SE5 9RS UK; 3 School of Immunology and Microbial Sciences, Faculty of Life Sciences and Medicine, King’s College, James Black Centre, 125 Coldharbour Lane, London, SE5 9NU UK

**Keywords:** Chronic liver disease, Cirrhosis, Multi-drug resistant organism, Antibiotic resistance, Antibiotic stewardship, Rapid diagnostic tests, Resistome, Immune modulation, Faecal microbial transplantation

## Abstract

High levels of antimicrobial drug resistance deleteriously affecting the outcome of treatment with antibacterial agents are causing increasing concern worldwide. This is particularly worrying in patients with cirrhosis with a depressed immune system and heightened susceptibility to infection. Antibiotics have to be started early before results of microbiological culture are available. Current guidelines for the empirical choice of antibiotics in this situation are not very helpful, and embracing antimicrobial stewardship including rapid de-escalation of therapy are not sufficiently emphasised. Multi-drug resistant organism rates to quinolone drugs of up to 40% are recorded in patients with spontaneous bacterial peritonitis on prophylactic antibiotics, leading to a break-through recurrence of intra-peritoneal infection. Also considered in this review is the value of rifaximin-α, non-selective beta-blockers, and concerns around proton pump inhibitor drug use. Fecal microbial transplantation and other gut-targeting therapies in lessening gut bacterial translocation are a promising approach, and new molecular techniques for determining bacterial sensitivity will allow more specific targeted therapy.

## Introduction

Ten million lives every year and a cumulative USD$ 100 trillion of economic output are estimated to be at risk by 2050 due to the rise of multi-drug resistant organism (MDRO) infections, with over 700,000 people currently dying each year as a direct consequence of resistant infections [1]. MRDO is defined as resistant to three or more antibiotic classes, including β-lactams [2]. The relentless rise in emergence of MDRO affecting patients with chronic liver disease (CLD)—defined by the presence of cirrhosis—is of worldwide concern, with an overall global prevalence of 34% and MDR rates highest in Asia, particularly India, and in South America [3]. In Europe, a recent study of cirrhosis patients showed the prevalence of MDR to have increased from 29 to 38% in culture-positive infections over the period 2011–2018 [4].

In a recent large multi-centre prospective intercontinental study to assess the prevalence and outcomes of bacterial and fungal infections in patients with cirrhosis [3], MDR and extensively drug-resistant (XDR) bacterial infections were very common on the Indian subcontinent (73% and 33% of isolates, respectively), whereas the prevalence of MDR infections was lower in North American centres (27% and 4% in United States, respectively), with significant variability across Europe (from 57% in Israel to 17% in Russia). Patients with MDRO infections had a higher incidence of septic shock, need to be transferred to the intensive care environment, and need for mechanical ventilation or renal replacement therapy than those with non-MDRO infections. Length of hospital stay was significantly longer in patients with MDRO infections than in those without. Those with MDRO infections had a significantly higher in-hospital mortality rate, with a greater cumulative incidence of mortality at 28 days (29% vs 20%; *p* = 0.014).

It is important to consider wider practices outside of the delivery of healthcare that contribute to the development of MDRO. Environmental antibiotic pollution due to industrial waste or used in the animal sector encourages the transfer of resistance genes in those bacteria found in humans, which often have a pathogenic role [5]. Countries where there is suboptimal or no regulation of such practices such as India further compounds these problems, but also presents an opportunity to begin to combat not only this environmental threat [6], but also that encountered due to a lack of regulation of antibiotic dispensing and direct sale to the consumer [7, 8] which enables antibiotic misuse and further encourages development of AMR. Wastewater treatment plants that serve antibiotic manufacturing facilities in India are implicated in the transfer of AMR into human microbiota, a problem that is magnified not only because of the size of the pharmaceutical sector in the subcontinent [9], but also because of a lack of government regulation around the discharge of such waste into the environment. Antibiotics are used increasingly as growth promoters on the Indian subcontinent (and elsewhere globally) as the demand for poultry and meat grows inescapably, which positively selects pathogens with AMR potential [10]. In other parts of the world in addition to India, including Brazil, Russia, China and South Africa, the increase in antimicrobial consumption by livestock will be 99%, which is up to seven times the projected population growth in this group of countries, further increasing the selection pressure on bacteria to become resistant [10]. Studies in various regions of India have confirmed the presence of antimicrobial residues in chicken meat and milk, indicating the widespread use of antimicrobial use in the food chain [11]. An in-depth review of these environmental, agricultural and industrial impacts on AMR development can be located on the World Health Organisation website [12].

Restricting the inappropriate use of antibiotics—the cornerstone of approaches to reduce MDR—is particularly difficult in CLD patients, with their known increased susceptibility to infection as a result of excessive intestinal microbial translocation [13, 14] and deranged immune function [15]. Acute bacterial infections are often the immediate cause of death, associated with a 400% increase in mortality in hospitalised patients, and a post-infection mortality rate of 28% and 63% at 1 month and at 1 year, respectively [16, 17]. In decompensated cirrhosis, the rate of infection is disproportionally high at 34% per year in patients and in up to half, infection is the cause of hospital admission, with over a third subsequently developing nosocomial infections, as compared to approximately 5–7% of the general population [18, 19].

Numerous carefully controlled studies have shown the necessity for the earliest possible start of antibiotic therapies for infective complications—pneumonia, urinary tract infections or spontaneous bacterial peritonitis (SBP)—if the patient is to survive. A study of cirrhotics with septicemia showed that each hour of delay in starting antimicrobial therapy resulted in an almost doubling of hospital mortality [20]. Patients bleeding from oesophageal varices also have much better survival, with less re-bleeding, if treated early with antibiotics. These data, however, are derived from largely single-centre studies and have been applied to those at any stage and severity of liver disease—even mild. This has led to antimicrobial over-prescription which contributes to MDRO development [21]. Similarly, patients with SBP need to be started promptly on antibiotics at the time of diagnosis if progression to septicemia is to be avoided. Infections are also of major importance in precipitating progression of stable and decompensated cirrhosis to acute-on-chronic liver failure (ACLF), where multiple organ failure occurs and a much higher mortality [4].

In none of these instances, is it possible to wait for 24–48 h for the result of a bacterial culture and antibiotics have to be started empirically on the basis of the best guidance available. Patient outcomes are inevitably affected deleteriously by the occurrence of MDRO, where the choice of initial empirical therapy is insufficient. In one study of SBP patients on prophylactic quinolones, 45% had on culture bacteria resistant to the first-line empirical choice [21]. Furthermore, cirrhotic patients are highly susceptible to infections driven by MDROs because risk factors for developing multi-resistance concentrate in this population in relation to (1) their inherent susceptibility to infection, (2) repeated hospitalisations particularly when decompensated, (3) the need for invasive procedures, often repeatedly, (4) subsequent frequent acute and prophylactic antibiotic exposure which positively selects for MDRO, and (5) depending on geographical region, the huge variation in the use of empirical antimicrobial therapy including differential practices in narrowing the spectrum of antimicrobial action, de-escalation and cessation [22]. These factors and practices in combination result in cirrhotic patients developing AMR more readily and at ever increasing rates.

## Antibiotic stewardship and effective use of empirical and antibiotic prophylaxis; current guidelines

Evidence of a reduction in AMR rates in cirrhosis as a direct consequence of antimicrobial stewardship programmes is limited. Whilst the rationale for such programmes remains strong and logical [12, 20, 23], the challenge for future studies is to apply more robust design and evaluation when assessing behavioural change interventions [12], and how this translates into clinically and microbiologically relevant outcomes. Current evidence in the critical care environment shows that antimicrobial stewardship is associated with improved antimicrobial utilisation and does correspond with improvements in AMR rates and adverse events, without impacting adversely on short-term clinical outcomes [13]. If similar programmes can be effectively implemented in the care of cirrhotic patients, a reduction in AMR rates would be anticipated.

The prescribing guidelines that exist are based on variable levels of evidence [24]. These make only broad recommendations around the need to obtain representative samples early for microbiological culturing and use of broad-spectrum antibiotics for maximal empirical coverage. Empirical treatment should be based on local microbiological susceptibility data, given that bacterial infection patterns and AMR rates vary very significantly geographically from treating centre to centre, and region to region. Such region-specific guidelines are currently lacking. Large variation in not only the types of infections but also MDR rates was demonstrated in two recent large scale studies which reported the incidence of MDR infections in cirrhotic patients in Europe [25] and globally [3]. Whilst empirical therapeutic regimens are recommended in recent European guidelines for different types of infections in cirrhosis, these highlight the importance of choosing initial antibiotics based not only on the type, severity and suspected origin of infection (community-acquired, nosocomial or health care-associated) but importantly also on local epidemiological data of antibiotic resistance profiles [19, 24].

Little attention is given to the need for rapid de-escalation and how this could be achieved by reducing the coverage of the initial broad-spectrum empirical drug, by switching to a narrow-spectrum antimicrobial, and active and early withdrawal of antibiotics when infection resolves or is not confirmed in the first instance. De-escalation—pivotal to antibiotic stewardship [26]—is thought to be vital to reduce the inappropriate and overuse of antimicrobials which drives the development of MDRO. Furthermore, standard microbiological techniques are still unable to identify clinically relevant infection-causing organisms, compounding the difficulties around narrowing the drug spectrum, and identifying sensitivity patterns. Specific biomarkers to aid in either the earlier detection of infection or to guide de-escalation of antimicrobial therapy in cirrhosis are currently difficult to recommend, with none to date being of satisfactory value by way of representativeness and severity of infection, accuracy or reproducibility [15, 19]. Whilst examples such as C-reactive protein, procalcitonin, lipopolysaccharide-binding protein and soluble CD14 are well established acute-phase proteins that have been investigated in several experimental and mechanistic contexts in cirrhosis, their utility in decision making in the acute clinical setting remains controversial and requires further evaluation.

Duration of antibiotic therapy for both acute infections and for prophylaxis are discussed in detail in European guidelines focusing on the treatment of bacterial infections in cirrhosis [19], and as part of the management of patients with decompensated cirrhosis [24]. These guidelines may not, however, be fully applicable to other parts of the world, such as in Asia and parts of South America, where AMR profiles are higher and different as evidenced by the recent global study [3]. Duration of antibiotic treatment—including for ‘culture-negative’ sepsis—has not been formally investigated or defined in cirrhosis, except for spontaneous bacterial peritonitis with a minimum of 5 days recommended [27]. Routine prophylaxis is currently recommended only for patients that are at the highest risk of developing bacterial infections, namely those with spontaneous bacterial peritonitis (SBP), low-protein ascites and episode of variceal hemorrhage (Table 1) [24]. Where cirrhotic patients are prescribed antibiotic prophylaxis for the primary or secondary prevention of SBP, these patients may be exposed to these long-term antibiotics for months or even years, until the occurrence of liver transplantation or death. This very extended duration of antibiotic therapy paradoxically significantly increases the selection pressure for and risk of development of MDRO in these patients, potentially driving the development of AMR.

**Table 1:** Current indications and recommendations for antibiotic prophylaxis in cirrhosis (adapted from [24])

Indication	Antibiotic and dose
Variceal bleeding	Preserved liver function: norfloxacin 400 mg/12 h orally for 7 days Decompensated cirrhosis (at least 2 of: ascites, jaundice, hepatic encephalopathy, malnutrition): IV ceftriaxone 1 g/day for 7 days
Primary prophylaxis of spontaneous bacterial peritonitis (SBP) in patients with low protein ascites (< 15 g/L)	Norfloxacin 400 mg/day orally or ciprofloxacin 500 mg/day until transplantation or death with decompensated cirrhosis Child–Pugh score ≥ 9 points with serum bilirubin ≥ 51 mmol/L and/or Renal dysfunction (serum creatinine ≥ 106 µmol/L, blood urea nitrogen ≥ 8.92 mmol/L and/or serum sodium ≤ 130 mmol/L)
Secondary prophylaxis of SBP	Norfloxacin 400 mg/day orally until liver transplantation, death, resolution of ascites or improvement in liver function to compensated state

Antibiotic prophylaxis in CLD is currently centred on the use of fluoroquinolone class of antibiotics, such as norfloxacin and ciprofloxacin, which are active against both Gram-negative and Gram-positive bacteria. These target the most common organisms implicated in spontaneous infections [28] acting locally within the gut, and in doing so prevent translocation of these bacteria and exposure to their immunologically activating virulence factors across the dysfunctional intestinal epithelial barrier. Emerging data from Europe confirm a rise in SBP episodes caused by Gram-positive and MDRO [29], lowering the effectiveness of the current internationally recommended first-line antibiotic regimens, translating into worsening prognosis and an increase in in-hospital mortality.

### Fluoroquinolones

In a recent placebo-controlled multi-centre trial where norfloxacin was used to treat 291 patients with Child–Pugh C cirrhosis for a total of 6 months [30], norfloxacin significantly decreased the incidence of any and Gram-negative bacterial infections, without increasing infections caused by *Clostridium difficile* or MDRO. However, only patients who had not received fluoroquinolones within the past month were eligible to be included in the study which undermines the results and extrapolation to real-world settings. This is because CLD patients with ascites have a high probability of being exposed to this class of antibiotics given current recommendations for SBP treatment [24, 31], and the broad spectrum of activity of fluoroquinolones combined with a high frequency of mutations in the target bacterial enzymes have also been shown to alter the bacteriology of SBP infections, with a high prevalence of gram-positive bacteria and extended-spectrum β-lactamase-producing *Enterobacteriaceae* [32]. The intestinal-specific action of quinolones, therefore, means that there are major concerns in whether this drives the selection of AMR genes in the gut microbiome of these patients. In the aforementioned retrospective cohort study conducted in Buenos Aires, Argentina of 115 patients receiving norfloxacin for the secondary prophylaxis of SBP, the 1-year cumulative incidence of SBP recurrence in CLD patients despite secondary prophylaxis was high at 28.5% [32]. Given that as many as one third of patients receiving norfloxacin as prophylaxis may still experience SBP recurrence, alternative antibiotic and non-antibiotic based prophylactic strategies require urgent evaluation [28, 33].

Another recent study of cirrhotic in-patients from the USA compared outcomes for those on primary *vs* secondary SBP prophylaxis, where almost three quarters were on norfloxacin and the remainder on trimethoprim–sulfamethoxazole [34]. The two groups were propensity-matched for MELD score and serum albumin during the index admission and 90-day follow-up (154 in each group). Patients receiving primary prophylaxis for SBP paradoxically had worse outcomes than those on secondary prophylaxis, who were more likely to have refractory ascites, multiple hospitalisations within the prior 6 months and more difficult to control hepatic encephalopathy (HE). In particular, primary prophylaxis patients had a higher mortality (35% vs 22%; *p* = 0.02) compared to secondary prophylaxis patients. Those on secondary prophylaxis were, however, more likely to still develop SBP (10% vs 22% *p* = 0.004), and worryingly this group had a higher rate of Gram-negative bacterial infection, which are the organisms that fluoroquinolone-based prophylaxis should prevent, with the likelihood that such prophylaxis is in fact selecting for resistant organisms and further driving the development of AMR.

It should also be noted that the extended use of fluoroquinolones as prophylaxis can cause significant adverse events. The United States Food and Drug Administration (FDA) previously issued enhanced warnings referring to disabling and potentially permanent side effects involving tendons, joints, muscles, and the central nervous system [35]. In July 2018, the FDA strengthened its warning for fluoroquinolones, including a separate notice about side effects potentially affecting mental health (disturbances in attention, disorientation, agitation, memory impairment and delirium) and the risk of hypoglycaemia-induced coma. Despite this, the FDA maintains that “the use of fluoroquinolones has a place in the treatment of serious bacterial infections where the benefits of these drugs outweigh the risks, and they should remain available as a therapeutic option” [36].

### Rifaximin-α

The antimicrobial drug rifaximin-α, which is > 99% non-absorbed from the gut, received regulatory approval not to treat or prevent bacterial infections in cirrhosis, but as secondary prophylaxis against overt HE. Whilst rifaximin-α has been shown in multiple randomised controlled trials to be clinically effective in this particular setting, the underlying mechanism of action has been linked to not only an anti-microbial effect within the gut, but also via other biological pathways involving gut microbiota functional modulation, inflammation attenuation via the pregnane X receptor, and immunological reconstitution [37, 38].

AMR to rifamycin—which includes rifaximin-α—was initially thought to be uncommon but due to a simple mutation in the *rpoβ* gene which codes for rifaximin-α’s molecular target, RNA polymerase β subunit [39], there are now real concerns around the implications of this in a cirrhotic population. Rifamycin-resistant *Clostridium difficile* infection (CDI) causing strains have mutations in the *rpoβ* gene, and these mutations also underlie resistance to both rifamycin and rifampicin when used in anti-mycobacterial therapy.

Resistance to rifaximin-α was shown to appear rapidly in cirrhotic patients treated for HE in an outbreak of CDI caused by ribotype 027 (B1/NAP1) [40]. 22% of affected patients had underlying cirrhosis. Recurrence of CDI-027 was significantly higher in cirrhotics on rifaximin-α (44.4% vs 14.8%). Another study of 388 cirrhotic patients reported *C. difficile* resistance to rifaximin-α of 34.1% overall and 84.6% in patients who had previously received rifaximin-α [41]. The widespread use of rifaximin-α in the USA for HE, as well as other indications such as irritable bowel syndrome, has coincided with a marked rise of resistance from 8% in 2006–2007 to 35% in 2011 in a university hospital in Texas with a liver transplant programme [42]. What is alarming is that infection by rifamycin-resistant strains of *C. difficile* was not shown to relate to prior use of rifaximin-α or to acquiring the infection in the hospital, suggesting a more widespread resistance profile not necessarily related to prior drug exposure.

## Impact of non-antimicrobial therapies on infection development and AMR risk

Other pharmacotherapies commonly prescribed in the management of cirrhotic patients also have the potential to have either a positive or detrimental impact on the subsequent development of infection and thus AMR. Here we consider the implications of use of non-selective beta-blockers (NSBB) and proton pump inhibitors (PPI).

### Non-selective beta-blockers (NSBB)

Propranolol, carvedilol and other NSBBs—widely used in the management of portal hypertension—have sympatholytic effects that may play an important role in reduction of bacterial translocation and by increasing bowel motility, with an improvement in intestinal permeability evidenced by a reduction in lipopolysaccharide-binding protein and interleukin-6 in the plasma [43] and in vascular endothelial dysfunction [44]. Indeed, a meta-analysis performed on four studies demonstrated a significant difference in favour of NSBB in preventing SBP [45] and in a study of cirrhotic patients with refractory ascites awaiting liver transplantation, use of NSBB was independently associated with reduced mortality (adjusted HR 0.35, *p* = 0.022) [46].

A previous study of 245 patients with refractory ascites but without infection, taking NSBB, reported a significant reduction in hospitalisation [47]. At multivariate analysis, NSBB treatment correlated with higher transplant-free survival (HR 0.771; 95% CI 0.598–0.993; *p* = 0.04). A correlation was reported between mortality and NSBB *only* in patients experiencing a previous episode of SBP, with a significant reduction in transplant-free survival of the SBP experiencing cohort (HR 1.644; 95% CI 1.145–2.361). These data where the majority of patients had Child–Pugh C cirrhosis suggest that NSBB negatively influence hemodynamic status in patients with infection, but not that NSBB therapy even in advanced cirrhosis represents a risk factor for developing infection.

NSBB target the pathophysiological pathways that propagate portal hypertension, and their use might also extend to having beneficial non-hemodynamic pleiotropic effects [48] within a therapeutic window based on stage of cirrhosis that remains controversial and needs to be defined [49]. Their use has been demonstrated recently to not only reduce variceal hemorrhage for which they are primarily prescribed but in compensated cirrhosis to also increase decompensation-free survival in patients with clinically significant portal hypertension [50]. This effect is mainly by reducing the incidence of ascites, and there are likely to be additional mechanistic effects in relation to bacterial translocation which remain to be elucidated.

### Proton pump inhibitors (PPIs)

PPIs are also widely prescribed in patients with cirrhosis and have been associated with an increased incidence in infection-related complications such as CDI and SBP in CLD, and whether this drives the development of AMR. This has been linked to a reduction in the diversity of gut microbiota and outgrowth of pathogenic species [51]. A study involving 1827 healthy twins found a significant decrease in alpha-diversity and alteration in bacterial composition in the PPI users, with a higher abundance of oral bacteria, including Streptococcaceae [52]. Removal of the low pH barrier by inhibition of gastric acid secretion reduces the ability to filter out oral and upper GI bacteria allowing migration unchallenged to the lower gut, colonising and predisposing to small and large intestinal dysbiosis and enteric infections [53]. The phenomenon of distal migration of oral bacteria in cirrhotic patients has been reported where 54% of the 28 patient-enriched, taxonomically assigned species detected in the feces were of buccal origin [54]. This was reconfirmed in a study where a microbiota shift and functional change in the distal gut in patients with compensated cirrhosis was demonstrated [55], suggesting that this could set the stage for bacterial overgrowth and heightened infection risk.

A 5-year follow-up observational study assessed the impact of long-standing PPI use on outcomes in a cohort of 350 cirrhotic patients, divided between regular PPI users (*n* = 196) and non-users (*n* = 154) [56]. Regular PPI use was associated with an increased cumulative probability of developing SBP compared to non-users [55% vs. 24.8%, hazard ratio (HR) 4.25; *p* = 0.05]. A similar association was found between regular PPI use and risk of first hepatic decompensation (HR 2.81, *p* = 0.008, *n* = 146) in previously compensated patients, and increased liver-related mortality (*p* < 0.001). Regular PPI use (HR 2.81, *p* = 0.003) and MELD score (HR 1.21, *p* < 0.001) were independent predictors of mortality, with the authors speculating that PPI use enhanced bacterial translocation which accelerated the development of hepatic decompensation and death.

## Molecular and other diagnostic techniques for rapid identification of infecting organisms and AMR gene profiling

Newer technologies for determining antimicrobial susceptibility rapidly offer the potential to speed up the clinical administration of an appropriate antibiotic regimen and/or de-escalation, and will increasingly be key to the successful implementation of antimicrobial stewardship programmes [57–59]. These include (1) MALDI-TOF (matrix-assisted laser desorption/ionization time-of-flight) mass spectrometry [60], (2) automated combined bacterial (and fungal) identification within 90 min and antimicrobial susceptibility in approximately 7 h via Accelerate Pheno platform [61], and (3) nanotechnology partnered with microfluidics [62]. Other techniques are based on micro-arrays or multiplex PCR platforms capable of detecting gene targets specific to MDROs.

‘Point-of-care’ diagnostic testing such as the MinION™ device manufactured by Oxford Nanopore Technologies has the potential to allow real-time microbial DNA and RNA sequencing using a portable device that can be used in the clinical environment [63]. MinION characterises bases on a bacterial DNA strand by measuring changes in electrical conductivity generated as they pass through a biological nanopore. This technology is fully portable and requires no additional computing infrastructure making it suitable for application at the beside. Another platform is the Curetis Unyvero™ system which employs cartridge technology that can detect not only over 100 different pathogens in a single cartridge within 4–5 h, but can also provide data on sixteen different AMR genes even in polymicrobial infections to antibiotics classes such as aminoglycosides, macrolides, 3rd generation cephalosporins and carbapenems. In addition to blood, the system can handle complex samples such as viscous sputum, broncho-alveolar lavage, tracheal aspirates, synovial fluids, feces and urine. It enables sensitive multiplexed testing, with initial DNA isolation and purification, followed by a specific multiplex PCR amplification step with final DNA detection optimised for hybridisation within a few minutes. Current and relevant barriers to implementation of such systems include a relative lack of robust and clinically relevant data of their utility in the setting of CLD and cost. As these factors begin to be addressed within the wider strategy of tacking AMR in CLD, we envisage these technologies being embraced and implemented, particularly in the critical care environment where polymicrobial infections are common and rapid diagnosis, identifying susceptibility profiles and expedient administration of effective antimicrobials are vital steps in improving survival [20].

A separate strategy to characterise the antibiotic resistance genes harboured by bacteria—known as the ‘resistome’—provides valuable insight into mechanisms around the development of MDROs. This is of particular relevance to the human gut, given that this is where 95% of all microorganisms detectable in the body by way of the gut microbiota are resident [64]. This densely populated microbial ecosystem resident in the intestinal luminal environment provides frequent opportunity for the horizontal transfer of resistance genes amongst microbes, through several different mechanisms including conjugation and transduction, with most AMR genes harboured by strictly anerobic intestinal commensals. Facultatively anerobic bacteria, in particular, those that produce lactic acid such as enterococci, streptococci and lactobacilli, are also involved in horizontal gene transfer within the gut [65]. This is particularly relevant to cirrhosis because enterococci, which are known to be enriched in the feces of patients with CLD, appear to behave as efficient ‘drug resistance gene traffickers’ in the gut [66], and thus may have an impact on development of enteric AMR, with emerging data confirming increasing prevalence of MDRO including vancomycin-resistant strains [3, 25, 67].

To understand the range of different resistance genes that allow bacteria within a particular anatomical niche to withstand antibiotic therapy, the entire microbiome has to be interrogated. Given that most bacteria cannot be cultivated in the laboratory even under the most optimal and adapted conditions, the reservoir of antibiotic resistance genes in the traditionally non-cultivatable majority remains relatively unexplored. Evaluation of the resistome and complex antimicrobial resistance gene profiles is possible via shotgun metagenomic sequencing (MGS) [68]. These resistance genes can then be mapped against established and evolving resistance gene databases to characterise the resistome, such as the Comprehensive Antibiotic Resistance Database (CARD) [69], Resistance Determinants DataBase (RED-DB) [70]‚ ResFinder [71], ARG-ANNOT [72] and Resfams [73]. These data will improve the understanding around how abundant these AMR genes are in the gut microbiome of cirrhotic patients, how this impacts on the subsequent development of MDRO infections and clinical outcomes, and importantly, aid in developing therapies to target these pathways.

Differentiating the complex systemic inflammatory response to active microbial infection from underlying excessive sterile inflammation related to cell death and release of damage-associated molecular patterns in advanced cirrhosis [74, 75]—which can both result in clinical deterioration manifesting particularly with organ failure—remains a challenge, hence the need for accurate diagnostics. Assessment requires an individualised and thorough approach to each patient, utilising all available information gleaned from the presenting history, physical examination, standard laboratory and other microbiological parameters [24]. This makes the case even stronger for implementation of rapid molecular-based diagnostic techniques to help confirm the presence (and indeed absence) of infective pathogens, the likelihood of AMR and aiding in selecting the best possible antibiotic regimen that takes resistance profiles into consideration as early as possible in the acute treatment of the cirrhotic patient.

## Systemic immune modulation improving the resistance of cirrhotic patients to infection: current and future perspectives

There are multiple potential avenues for systemic immunomodulation in CLD, many of which are at an early phase of investigation [76, 77]. Should these strategies have the desired effect of improving cirrhosis-associated immune dysfunction (CAID) [15, 78] and heightening the cirrhotic patient’s barrier to infection, this would require less exposure to antimicrobial therapy and, therefore, reduce the risk of developing AMR in the first instance.

Pre-/pro-/synbiotics, fecal microbial transplantation (FMT), prokinetics, FXR agonists as well as NSBB already referred to, all represent a means of targeting the gut-liver axis [79, 80] by differentially modulating gut microbial dysbiosis and reducing pathological bacterial translocation and, therefore, enterically derived infections as potential alternatives to traditional antibiotic use. Prokinetics and NSBB also improve intestinal motility, while bile acids and FXR agonists may help by improving intestinal barrier integrity, all of which are impaired in CLD.

Probiotics have been rationally proposed as a way of modulating the gut microbiome [81, 82] and have been trialled in cirrhosis, with varying degrees of success, in part due to the lack of robustly designed placebo-controlled randomised clinical trials [83]. Various types of probiotic therapies have been considered or trialled in NAFLD [84], HE [85–87], stable cirrhosis [88, 89] and decompensated cirrhosis [90, 91], with a variety of clinical and mechanistic endpoints. Results are conflicting in part due to variation in trial design and choice of endpoint(s), as well as the probiotic therapy used. Many of these trials are based on the use of a freeze-dried bacteria such as VSL#3, or single strain preparations such as Yakult™ which contains *Lactobacillus casei* Shirota, both of which may represent preparations that are suboptimal in the setting of CLD based on viability and potency.

FMT is known to be beneficial in non-cirrhotic patients who develop recurrent *Clostridium difficile* diarrhea. Trials in cirrhosis recently reported include a phase 1 safety study where cirrhotic patients with hepatic encephalopathy were randomised to either continue with standard of care alone or to also receive five days of antibiotics prior to a single FMT enema from a rationally selected donor [92]. Partial recovery of microbiota, bile acid and short-chain fatty acid profiles for patients in the FMT arm were reported, that were initially disrupted by antibiotic therapy, although the influence of the initial antibiotic course cannot be discounted. There are ongoing studies which focus on the utility of FMT in cirrhosis—such as PROFIT (EudraCT 2017-003629-13)—which for now continue to focus on safety, feasibility and tolerability [93].

Granulocyte colony stimulating factor (G-CSF) and statins are other emerging therapeutic strategies that have been shown to improve immune dysfunction in cirrhosis [94] and may indirectly affect the need for antibiotic therapy. However, the most recent trial of G-CSF with or without hemopoietic stem-cell infusion did not improve liver function and was associated with an increased frequency of adverse events when compared with standard care in a recent RCT [95]. Other immunorestorative strategies in cirrhosis remain at an investigative stage and are covered in detail elsewhere [77]. High-mobility group box 1 (HMGB1) which signals hepatocyte death and initiates local proinflammatory responses [96, 97], and MER receptor tyrosine kinase (MERTK) which is an important negative regulator of proinflammatory responses expressed on monocytes/macrophages [98] are two examples of molecular targets that may benefit from modulation.

Albumin administration has been recommended as part of the treatment for CLD patients diagnosed with SBP. Albumin (20%) infusion—in addition to its oncotic properties—has been shown to restore macrophage function by binding excessive free circulating prostaglandin‐E2 in patients with acutely decompensated cirrhosis [99]. A large scale RCT of long term albumin administration that involved 431 cirrhotic patients with ascites demonstrated an overall survival benefit with a 38% reduction in the mortality hazard ratio over 18 months [100] and a reduction in the incidence rate of bacterial infections, both SBP and non‐SBP related. Another single‐centre study of long‐term albumin administration [101] reported 2 year mortality was reduced (41.6% vs 65.5% in the SOC group) and was accompanied by significant reduction in SBP and bacterial infections other than SBP. Despite these positive data, long-term albumin administration is yet to enter into regular clinical practice, with clinical trials still in progress.

## Summary

**Fig. 1:**
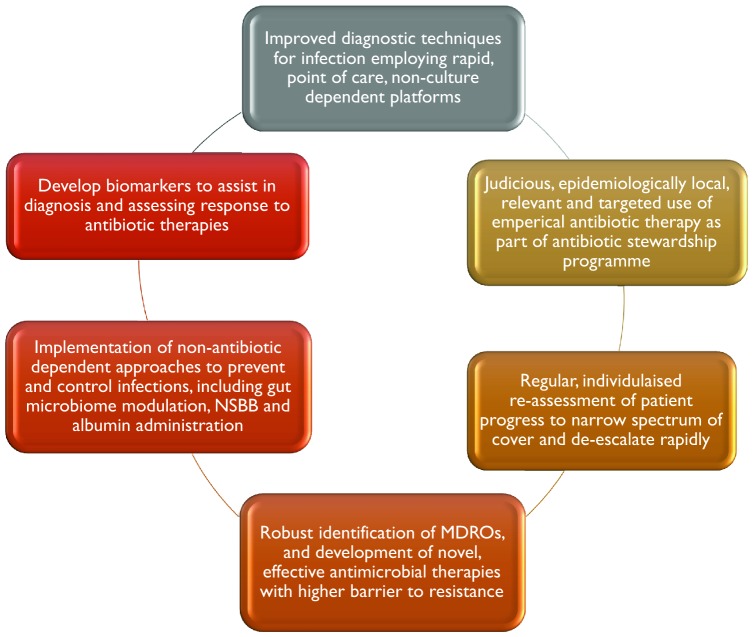
Strategies for effective antibiotic use and combating AMR in cirrhosis

Cirrhotic patients are particularly susceptible to bacterial infections, where their consequences are amplified due to systemic immune inadequacy and the propensity to develop organ failure. These observations have led to widespread and often inappropriate antimicrobial use, which is identified as driving the ever-increasing rates of AMR. It is clear that antibiotic stewardship programmes must form part of the effort to combat the rise in AMR. Specific advice tailored to local AMR prevalence is required to guide clinicians in the appropriate use of empirical antibiotics, encouraging narrowing of spectrum of activity as early as possible and then rapid de-escalation. Confidence in instituting these measures will require concerted investment in accurate and relevant biomarkers of infection onset and resolution, enhanced point of care, non-culture dependant molecular diagnostics for the rapid identification of infecting organisms, and the detection of resistance genes (Fig. 1). Further research is urgently needed to re-purpose existing therapies and develop alternative, non-antibiotic dependant immunomodulating strategies to increase the resistance of cirrhotic patients to infection in the first instance and define their optimal use either singularly or in combination with antibiotics.
